# Patients’ views on the use of an Option Grid for knee osteoarthritis in physiotherapy clinical encounters: An interview study

**DOI:** 10.1111/hex.12570

**Published:** 2017-08-04

**Authors:** Katharine Kinsey, Jill Firth, Glyn Elwyn, Adrian Edwards, Katherine Brain, Katy Marrin, Alan Nye, Fiona Wood

**Affiliations:** ^1^ Pennine MSK Partnership Ltd Greater Manchester UK; ^2^ The Dartmouth Institute for Health Policy and Clinical Practice Dartmouth NH USA; ^3^ Division of Population Medicine School of Medicine Cardiff University Cardiff UK

**Keywords:** decision support interventions, health literacy, qualitative research, shared decision making

## Abstract

**Background:**

Patient decision support tools have been developed as a means of providing accurate and accessible information in order for patients to make informed decisions about their care. Option Grids^™^ are a type of decision support tool specifically designed to be used during clinical encounters.

**Objective:**

To explore patients’ views of the Option Grid encounter tool used in clinical consultations with physiotherapists, in comparison with usual care, within a patient population who are likely to be disadvantaged by age and low health literacy.

**Methods:**

Semi‐structured interviews with 72 patients (36 who had been given an Option Grid in their consultation and 36 who had not). Thematic analysis explored patients’ understanding of treatment options, perceptions of involvement, and readability and utility of the Option Grid.

**Results:**

Interviews suggested that the Option Grid facilitated more detailed discussion about the risks and benefits of a wider range of treatment options for osteoarthritis of the knee. Participants indicated that the Option Grid was clear and aided their understanding of a structured progression of the options as their condition advanced, although it was not clear whether the Option Grid facilitated greater engagement in shared decision making.

**Discussion and conclusion:**

The Option Grid for osteoarthritis of the knee was well received by patient participants who reported that it helped them to understand their options, and made the notion of choice explicit. Use of Option Grids should be considered within routine consultations.

## 1 INTRODUCTION

Shared decision making (SDM) is a process in which patients are supported by clinicians within the consultation to consider treatment choices that are aligned with their informed preferences.^1^ Clinicians are required to elicit and respect patient preferences. There is a strong policy directive driving SDM in the United Kingdom,^2^, ^3^ which is mirrored in many other countries worldwide.^4^, ^5^ Patient decision support tools have been developed as a means of providing accurate and accessible information in order for patients to make informed decisions about their care. Evidence suggests that these tools given ahead of the consultation have a positive effect on increased patient knowledge,^6^ but there is very limited evidence to show that they can improve SDM within the consultation.^7^ However, there are studies showing that tools designed to be used *within the clinical encounter* can have a positive effect on the process of SDM.^8^

Option Grids^™^ are a type of decision support tool specifically designed to be used during clinical encounters. They are designed to orientate patients to the existence of choice by displaying treatment options side by side, organized by the concerns or questions that frequently occur to patients, thereby increasing the potential for SDM.^9^ The need for SDM is particularly relevant for patients with osteoarthritis of the knee, a common cause of physical disability in older adults that is associated with significantly reduced quality of life.^10^ The prevalence of osteoarthritis of the knee is predicted to rise in line with an ageing and increasingly obese population.^11^ Despite an increasing number of knee replacement operations being conducted in the UK,^12^ there is evidence of large unwarranted clinical variation which may be influenced by participants’ (lack of) awareness of the treatment options including knowledge and understanding of the risks and benefits of knee replacement surgery and significant differences being expressed by patients on whether surgery is the only reliable solution compared to others who wish to avoid surgery at all costs.^13^ Osteoarthritis of the knee can be managed by lifestyle measures such as improving activity levels to build strength in the joint and weight loss. Other management options include medication such as painkillers and anti‐inflammatories, steroid joint injections and knee replacement surgery.

Much of the research on SDM to date has been generated from trials where patient participants have had high educational and socio‐economic backgrounds. Concern has been expressed that decision support tools might exacerbate health inequalities as most are developed in English and may not be accessible to people with poor levels of health literacy or who do not have English as a main language.^14^ A recent systematic review has demonstrated that overall SDM interventions may benefit disadvantaged groups more than non‐disadvantaged groups, although interventions which were specifically tailored to disadvantaged groups were found most effective at achieving benefits.^15^ We had previously conducted a trial which demonstrated SDM increased when clinicians used the knee osteoarthritis Option Grid in consultations.^16^ The aim of this substudy was to explore patients’ views of the Option Grid encounter tool within a patient population who are likely to be disadvantaged by age and low health literacy, specifically eliciting patients’ views about how treatment options had been presented, and perceptions of their involvement in decision making.

## 2 METHODS

The current study reports data from interviews with patients recruited to a trial of the efficacy of an Option Grid decision support tool for osteoarthritis of the knee. The trial used a multiple interrupted time series (stepped wedge) design where patients were allocated sequentially to consult with an extended scope specialist physiotherapist who had consented to the trial. Patients are referred to the service from primary care and may then access a range of multidisciplinary specialist care for rheumatology, orthopaedics and musculoskeletal pain. Participating clinicians (physiotherapists) received brief (30 minutes) training on how to use the Option Grid within a routine consultation after having consulted with six patients using usual care procedures (the control group).

The Option Grid for osteoarthritis of the knee was developed by a team of clinicians and academics with an interest in SDM, musculoskeletal conditions and pain management. User testing informed its refinement.^17^ The Option Grid presents three recommended treatment options in a tabular format: pain killers, intra‐articular corticosteroid injections, and knee replacement surgery for symptoms refractory to non‐surgical treatment.^18^ Patients’ frequently asked questions are listed on the side of the grid, allowing readers to compare answers across the three treatment options. A copy of the grid is presented in Table 1.

**Table 1:** Option grid for Osteoarthritis of the knee. This grid is designed to help you and your clinician decide how best to manage your knee pain and activity level. The first steps are to become as fit and close to your ideal weight as possible and to consider having physiotherapy. Surgery is normally recommended only after other treatments have been tried 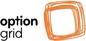

Frequently asked questions	Pain killers	Joint injections (steroids)	Knee replacement surgery
Will this reduce the pain I have in my knee?	It depends on which tablets are taken. Tablets like ibuprofen are effective for 50 in 100 people. Over the counter tablets, like paracetamol, including those that have codeine, are also effective.	Some people get good symptomatic relief after an injection, which may include pain relief and/or reduced swelling.	About 90 in 100 people who have this operation say it leads to relief of most or all of their pain, over time. 10 in 100 people say it does not lead to significant pain relief.
Will this treatment help improve which activities I can manage to do?	It may. As you get pain relief, you should be able to be more active and this in turn can also help to reduce pain. It helps to take painkillers before doing physical activity.	Yes, usually for up to a month or so. Plan to be more active as a result of the pain relief. Advice from a physical therapist may help.	Yes, the majority of patients experience improvement in their activity level. However, not everybody sees differences in their ability to walk or climb stairs.
Are there any risks to this treatment?	As with all medications, pain killers have some side‐effects. For example, codeine often leads to constipation and prolonged use of anti‐inflammatory tablets like ibuprofen increases your risk of stomach bleeding.	There is a small risk of frequent injections causing cartilage damage, especially in weight‐bearing joints. Allergic reactions and infections due to joint injections are uncommon. You might feel slight pain at the injection site for a few days.	Wound infection needing treatment occurs in 5 in every 100 people. Blood clots in the leg occur in 2 in 100 people. The risks from surgery increases if you have other conditions, such as heart or lung disease, are a smoker or are overweight.
How long will it take me to feel better after the treatment?	You may start experiencing pain relief within a few days of when you start taking the medication.	Most people who experience relief feel better within the first week or so after the injection.	Pain relief is gradual. You will stay in the hospital for around 3‐5 days. Most people walk unaided after 3 months. Full recovery usually takes between 6 and 12 months.
Will I need to have more treatment or surgery?	If things don't get better, talk to your clinician about other treatment options.	Pain relief lasts for up to a month or so. You can only have up to 4 injections per year.	Most knee replacements can last 15 years, many last longer.
What are the outcomes for people with arthritis who have this treatment?	Many people cope well by using medication, being active and losing weight. Reducing your pain may help you achieve the benefits of exercise.	Some people have good relief by having injections when swelling and pain cause problems.	Surgery is usually considered after other options have been tried. About 80 in every 100 people are satisfied after having a knee replacement. About 20 in every 100 are not satisfied.

Editors: Katy Marrin, Peter Alf Collins, Alan Nye, Mark Porcheret, Jo Protheroe, Victoria Thomas, Glyn Elwyn.

Evidence document: http://www.optiongrid.org/resources/osteoarthritisoftheknee_evidence.pdf.

More information: http://www.optiongrid.org/about.php.

Last update: 29‐Aug‐2012 Next update: 29‐Aug‐2013 ISBN: 978‐0‐9571887‐6‐1.

Creative Commons License: Attribution‐NonCommercial‐NoDerivs 3.0 Unported (CCBY‐NC‐ND 3.0).

Process and outcome measures were compared for patients participating in the intervention time period with those in the control time period.^19^ Ethical approval was obtained from the local research ethics committee (Ref: 11/WA/0356).

### 2.1 Participants and setting

Inclusion criteria for patient participants were adults with a diagnosis of osteoarthritis of the knee made by their GP or extended scope physiotherapist who were able to give informed consent. Potentially eligible patient participants were identified from referrals to a single musculoskeletal service that provides integrated services to the local population in Oldham, Greater Manchester, UK. A member of the clinical team attempted to telephone all eligible patients and invited them to participate in the study. Patients who expressed interest were sent an invitation letter, information sheet and consent form with their appointment letter. Informed written consent was given when they attended their appointment. Participants were recruited until the required sample size of 72 was reached (six physiotherapists consulting six patients each pre‐intervention and six patients each post‐intervention).^19^ In total, 78 participants were recruited, but six later were found to be ineligible or withdrew from the study. All 72 patient participants were interviewed. Clinicians were also interviewed and data from this are reported elsewhere,^20^ as are the main findings of the trial,^16^ in addition to a discourse analysis of how the Option Grids were used when an interpreter was present in the consultation.^21^

### 2.2 Data collection

For the purposes of the trial, a range of patient participant socio‐demographic variables were recorded including age, postcode, ethnicity, educational attainment and main language spoken. Patients were also asked to complete the REALM‐R reading exercise, which is a brief eight‐item measure of health literacy.^22^ Scores of 6 and under are considered to indicate poor health literacy. A research nurse (KK) was present in the consultation and observed the use of the Option Grid in the consultation and made structured field notes recording how the Option Grid was used during the consultation (number of questions asked, who held the Option Grid etc.). The consultation was also audiorecorded, but audiorecorded data and field notes are not presented in this paper. Short semi‐structured interviews with all patient participants were conducted immediately following the consultation in a private room by the same research nurse and were audiorecorded and transcribed verbatim. The aim of the interviews was to explore patients’ views about the consultation, including whether they felt involved in the decision‐making process, their views about how risks and benefits of treatment options had been presented to them, reasons for their prior and current treatment preferences, and their views about the use of the Option Grid within the consultation (if it was used). The interview schedule is outlined in Table 2 and was modified depending on whether an Option Grid was used and to allow the research nurse to incorporate her observations of the consultation. Data were collected over a period of 18 months.

**Table 2:** Interview schedule

1. Can you tell me about how the problems with your knee affect your life?
2. Can tell me about what you knew about the different treatment choices for your osteoarthritis of the knee before your consultation today?
3. Did you have any preference for the type of treatment you wanted for your osteoarthritis of the knee before you attended the clinic today?
4. I know I was in the consultation with you today but can you tell me what the person you saw today told you about the treatment options you could have for your knee?
Questions 5‐10 if an Option Grid was used:
5. How was the Option Grid used in the consultation?
6. What do you think about the way in which it was used?
7. What do you think of the way the different treatment choices for osteoarthritis of the knee were presented to you in the Option Grid?
8. How do you think the Option Grid affected the discussion you had with your clinician today?
9. You were given the Option Grid during your consultation, but some people have wondered if there might be a better time to give the Option Grid, perhaps before you come into the clinic, or as you are leaving the clinic? What are your views about this?
10. What do you think you will do with the OG when you get home?
11. Some people think that it is important that doctors are able to talk to their patients clearly and in a way that they understand. How well do you think the person you saw today communicated with you?
12. Do you think the person you saw today gave you enough information to help you make a choice about which treatment to choose?
13. Have you made a decision about treating your osteoarthritis of the knee?
14. Do you want more help from the person you saw today in clinic in making a decision about which treatment to choose?
15. What are your hopes for the future in relation to the pain in your knee?

### 2.3 Data analysis

A thematic analysis of the data set was undertaken as described by Braun and Clarke.^23^ This process started with KM familiarizing herself with the data, searching for meanings and patterns that captured something important about the data in relation to the aims of the study. This resulted in a set of initial codes. Given the large data set of 72 interviews, this initial familiarization and development of codes were generated from 20 interviews, which were from the first 20 patients enrolled in the study. A codebook^24^ was developed through joint discussion with FW. Following this, the remaining 52 transcripts were then coded by KM using the qualitative data analysis software NVivo10, and where necessary altering the code book to account for new subthemes. Attention was given to tensions and inconsistencies within codes and across data items where accounts were not consistent with the dominant story in the analysis. The codebook was refined as new themes emerged. KK then dual‐coded a 30% sample of the interviews. Discrepancies between the coders were discussed and resolved between members of the team. After all data had been coded, analysis focused on considering relationships between codes, how codes formed overarching themes and qualitative comparisons in coded data between patients who had used the Option Grid and those who had not used the Option Grid. Themes and subthemes were then reviewed to consider whether data coded at each theme formed a coherent pattern, and were then defined and named.

## 3 RESULTS

Table 3 presents characteristics of the 72 patients who were interviewed as part of the trial. Patient participants from each arm of the study were similar in terms of age and REALM‐R score, although there were differences in gender ratio between groups. The mean REALM‐R score for each group was slightly lower than the adult population mean of 6.8 (SD 2.1) found in a US validation study of the measure^22^; 29% of the participants had educational attainment at A’ level or above, compared to 39.5% for England and Wales as a whole.^25^

**Table 3:** Participant characteristics

Variable	Option Grid used	No Option Grid used
Mean age in years (range)	68 (42‐87)	64 (35‐82)
Gender (M:F)	11:25 (31%:69%)	18:18 (50%:50%)
REALM‐R score ≤ 6	8 (22%)	9 (25%)
Mean REALM‐R score (standard deviation)	6.1 (SD 2.65)	6.2 (SD 2.33)
Educational Attainment (A level or equivalent and above)	11 (30%)	10 (28%)
Non‐white ethnicity	4 (5%)	1 (1%)

We report the findings from the qualitative interviews grouped into three main sections which reflect grouped themes from the thematic analysis: (i) increased awareness of the treatment options, (ii) Option Grid is acceptable to patients and (iii) positive perception of involvement for most, but not all, patients. Data extracts are used to illustrate our data alongside participant identification number and socio‐demographic characteristics (whether an Option Grid was used, gender, REALM score and age).

### 3.1 Increased awareness of the treatment options

Participants who received the Option Grid appeared to demonstrate a greater awareness that there were a number of options available, although not all of the options were suitable for every patient:

He advised me on the three options…he said I wouldn't need the knee replacement unless it was a big downward trend on it and the agreement being to try the tablets and then the injections (P69, with OG, male, REALM 5, age 70)

In contrast patients who had consulted under usual care arrangements, without an Option Grid, appeared to be less clear that there were different treatment options that had been discussed during the consultation.

he explained it well and helped me to make me mind up that he was right, and me circumstances made me make that decision as well, so it was the only way I could go really. (P46, without OG, female, REALM 6, age 67)

Whilst participants in both groups felt that they understood the risks and benefits of treatments, participants who had used the Option Grid in the consultation demonstrated a greater awareness and understanding regarding the risks and benefits of treatment options. Some patients were able to accurately recall risks and benefits in reasonable detail, and much of this information was both significant and new to them:

It [the Option Grid] tells you about the injections only lasting for a limited period of time and it does say that there's a slight percentage of people that don't get a great deal of joy from a total knee replacement and you've got to consider these things… I thought there was a bigger percentage of failures for people who don't notice any significant improvement than what I thought. Before I came here today I thought that everybody that's had a knee replacement eventually was back to normal, but that's not the case. (P68, with OG, male, REALM 8, age 70).

Observation data also indicated that if an Option Grid was not used in a consultation, it was more likely that the clinician would focus discussion on the risks and benefits of the most likely treatment option. Consequently, patients were not made fully aware of the risks and benefits of the full range of treatment options. However, patients were not always critical of this strategy, rather they often recognized that this had occurred and attributed it to tailored information‐giving appropriate to their particular symptoms. When asked about whether she had been told the risks and benefits of knee replacement surgery, participant 20 indicated that she thought this was only relevant to the consent process rather than the decision‐making process:

No she didn't tell me. I suppose that would come… I should imagine like when you have a pre‐op they will tell you all the pros and cons then… they usually give you the percentage of the success rates as well. (P20, without OG, female, REALM 8, age 67)

From the patient interviews, it is difficult to ascertain clearly whether the Option Grid actually changed people's treatment preferences despite the interviewer asking whether the patient felt that the Option Grid had influenced their discussion and treatment decision. Often patients were not able to give a clear answer to the question of what their prior treatment preference was, instead stating that they wanted to get their knee “fixed” or receive some “wonder drug” (patient 74). Patients also understandably found it difficult to distinguish between the information given by the clinician and the information that they received due to the Option Grid being used in the consultation (any added benefit of the Option Grid). As patient 45 remarked:

Obviously it's just a matter of trying to pick the best choice for the situation….Yeah, he gives me some general information that possibly helped me in making the decision yeah. (P45, with OG, female, REALM 6, age 70)

There was, however, reflection amongst some patients that the Option Grid had helped to clarify and form their treatment decision.

do you think the Option Grid helped you make that decision? (Interviewer)

Probably – probably the Option Grid and listening to his advice as well – might seem the right step forward you know, it makes sense but like I said there's no quick fix and I think if I can lose the weight – and I am 3 or 4 stone over weight, if I can lose that and maybe just incorporate the mild painkillers you know I may be a lot happier in 4 months’ time. See how it goes. (P73, with OG, female, REALM 8, age 56)

### 3.2 Option Grid is acceptable to patients

Participants who had received an Option Grid expressed positive perceptions in relation to its content, reporting that it was clear and helped them to understand their options.

Although there were a few participants who suggested that the grid had provided them with enough, possibly more than enough, information.

Interviewer: What did you think about the level of information that was on there?

P24: Plenty, for my needs that is plenty of information. (P24, with OG, female, REALM 7, age 53)

Furthermore, many participants who had used the grid responded positively to the *purpose* (rather than the content) of the grid. A few commented that it and made the notion of choice explicit:

Lovely because I think it gives you basically – this is what you're asking for – the choice. (P78, with OG, female, REALM 8, age 73)

However, there were some indications that participants with very low literacy levels were not able to fully understand all the information presented in the Option Grid or understand the purpose of the grid.

it's alright. Yeah. It's fair. Just tells you the ins and outs of what you can have done so…I'm not the cleverest person but I should think a clever person would understand it a bit better (P32, with OG, male, REALM 2, age 53).

Most participants were also satisfied with the content and appropriateness of information provided on the Option Grid. However, a few participants thought there was insufficient information regarding analgesia and exercises/physiotherapy. In order to keep the Option Grid brief and easy to use, options such as podiatry and lifestyle changes were not included in this Option Grid, although clinicians were of course encouraged to discuss these options with patients regardless of whether they were using the Option Grid. Three participants were supported by a professional NHS interpreter during the consultation and interview, and all three reported that the Option Grid, verbally translated, provided useful information:

It gave me information which was good for me and I wanted the injection. (P33, with OG, female, age 82, REALM 0, with an interpreter).

However, the limitations for the three non‐English speaking patients in the study were clear as the same participant indicated that she would have no future use for the Option Grid, as she could not read it. In contrast, most participants whose first language was English felt the Option Grid would be a useful “take home tool” that they could refer to in the future and show family members or friends who had not attended the consultation with them.

Data from the interviews also suggested that patients who had been given an Option Grid were provided with more of a sense of a structured sequence of their treatment options as their disease course progressed, although some patients who had not received the Option Grid were also able to identify this staged approach:

I suppose it's elimination to see what works really and what doesn't work really and if it doesn't then go to the next level. (P56, without OG, REALM 7, male, age 61)

When asked if the Option Grid had been introduced at the appropriate time point within their patient journey, most participants felt that it had been timed correctly:

If I had had that [the Option Grid] before I came, I would have thought there's no other options, I'm stuffed, I have to do it [knee replacement surgery]. But, because I asked him questions about the recovery time and everything made me think differently. If you had given it to me afterwards I think that would've been a waste because he wouldn't have been there to ask questions. (P57, with OG, female, REALM 8, age 42).

However, some patients thought that there might be value in receiving a copy of the Option Grid before their consultation to help identify questions and discuss options during the consultation:

To go in the package you know with the – when I got all the information, I think that would be an option to put it in there to have a read prior to coming here and then we could have a discussion rather than sitting and leaving me to read it. (P66, with OG, male, REALM 6, age 46)

For one patient, the perceived delay in receiving the Option Grid in the consultation rather than prior to the consultation was expressed as frustration as she believed had she been better informed before the consultation then the consultation might have concluded with a treatment decision rather than a recommendation to reflect more on options.

I think definitely it would have helped an awful lot where as now I have to take it home and read it and I might have been able to make a decision and have discussed it and then I can have that treatment– so that definitely had I seen it before. (P55, with OG, female, REALM 8, age 87)

Participants were also able to reflect that although they had been given the Option Grid in the consultation, they, or members of their family, would be able to reread it and reflect on it after the consultation.

You know, when I get home I'd most probably read it completely properly again you know but as far as I'm concerned it did give you a lot of information. (P26, with OG, female, REALM 6, age 66)

### 3.3 Positive perception about involvement for most, but not all, patients

When asked whether the Option Grid promoted their involvement in decision making, most participants answered positively. However, most participants in both groups appeared to feel that they had made their own treatment decision, with guidance from the clinician, making it difficult to determine the added benefit of the Option Grid from patients’ reports of their involvement:

[Clinician] helped me make the decision but it was my decision at the end of the day (P76, with OG, male, REALM 6, age 45).

I think it [the decision] was mine, I think he, you know pretty much – here are the choices to go for this or this, he kind of did make it up to me really in the end its your decision. (P53, without OG, female, REALM not completed, age 58)

There were, however, occasions when participants from both groups adopted a passive stance and still felt it necessary to defer the decision to the clinician. This passivity towards involvement was particularly notable from patients who had low health literacy scores:

I'd leave it up to [Clinician] to sort out (P32, with OG, male, REALM 2, age 53).

I don't think I've got any option really…. If that's what I've got to do I've got to do it and that's the end then. (P46, without OG, female, REALM 6, age 67)

Most participants expressed a desire or need to involve others in decision making. Participants in both groups indicated that they would discuss the options with their primary care physician or members of their family, although it was not always clear how this extended advice seeking influenced the decision itself. One participant in particular was clear that he would need to discuss the surgical option with his wife as it would impact on both of them:

I do think about the family side of it as well. I wouldn't like to think that I've plumped for something that would put me out of commission for weeks and weeks (P68, with OG, male, REALM 8, age 70).

However there were two participants, both of whom were not given the Option Grid, and both had low health literacy scores, who did not feel their decision matched their treatment preference:

Well you have to listen to the experts, so I'll go along with him. I would have preferred the injection. (P40, without OG, male, REALM 2, age 64)

Well I think he [the clinician] thought that would be the best line to go through and I thought whatever advice he gave me I would go with that. I didn't want to say, ‘oh no, I want a knee replacement’ if he thought the injections would work. (P51, without OG female, REALM 6, age 72)

## 4 DISCUSSION AND CONCLUSION

### 4.1 Principal findings

Patients who received the Option Grid for osteoarthritis of the knee reported that it was clear, helped them to understand their options and made the notion of choice explicit. Our data also suggest that patients who received the Option Grid had a better understanding of the full range of treatment options. Participants who used the Option Grid provided positive feedback, with some suggestions around content such as the inclusion of exercise supported by physiotherapy in the tool. The Option Grid appeared to provide patients with a more detailed understanding of the risks and benefits of treatment options and, from the patients’ perspective, was an acceptable and useful tool that facilitated the conversation around treatment options within the consultation. However, it was not clear from the patient interviews whether the Option Grid facilitated greater engagement in SDM as both groups of patients reported that they felt involved in the treatment decisions, nor is there any evidence from the patient interviews that the Option Grid resulted in appropriate treatment decisions. Furthermore, there is some evidence that patients with low literacy struggled to engage with the Option Grid decision aid.

### 4.2 Strengths and weaknesses

Interviews were conducted immediately following the consultation and so patients would be expected to have reasonable recall of their experiences of their encounter. The data set of 72 interviews is comparatively large for a qualitative study and patients recruited were similar in respect to age and gender compared to all patients deemed eligible for the study and in respect to age and gender were typical of the population that is normally affected by osteoarthritis of the knee. Although the interviews were conducted by the research nurse, participants were encouraged to be candid in sharing their views about the consultation.

Lessons learned from a review of SDM implementation programmes suggest that local context is a vital factor in implementation.^26^ The study site has a history of partnership working to promote self‐care, providing skills and training in SDM and has the support of clinical leaders, which are factors associated with other local change programmes that have often fared better at implementing new models of patient‐centred care. Patients in the “usual care” group were therefore likely to be consulting with clinicians who were practising SDM to some degree. The addition of the Option Grid in the “intervention arm” of the study, as a means of structuring the discussion of treatment options, was therefore an adaptation to the way in which the physiotherapists had previously consulted.

The Option Grid only included three medical treatment options of medication such as painkillers and anti‐inflammatories, steroid joint injections and knee replacement surgery. Other self‐management options such as weight loss and lifestyle changes were not included in the grid, although clinicians did discuss the benefits of these self‐management options with patients. Had the self‐management options also been included in the Option Grid, then patients’ views about the grid and their involvement in decision making may have been different.

### 4.3 Comparison to other literature

There have been numerous studies which have examined patients’ views of participating in the decision‐making process including studies which have highlighted patient‐perceived barriers and facilitators to SDM (for a review, see Joseph‐Williams et al.^27^). Fewer studies have more specifically explored patients’ views of decision aids and generally these studies have found that patients welcome decision aids for their informational content.^28^, ^29^ A study conducted in the United States by Tiedje et al.^30^ of patients’ and clinicians’ experiences of using a decision aid within a consultation (what we call an encounter tool) identified that the decision aid created a space for discussion of treatment options. Our data also indicate that patients find encounter tools a useful and flexible scaffold around which to deliberate their options during the consultation. Data from this paper, which demonstrates patient support for the Option Grid, complement our main findings that use of the Option Grid increases the amount of SDM in the consultation.^16^ It also complements our data derived from interviews with clinicians (reported elsewhere^20^) which demonstrates that clinicians, although initially sceptical, became more confident that Option Grids were acceptable to patients, allowed them to discuss options with a more neutral approach and encouraged engagement from patients. Together, these substudies indicate support for the Option Grid from both parties within the clinician‐patient dyad.

Previous survey research has found that patients reported more discussion of pros than cons in relation to 10 common treatment decisions.^31^ Encounter tools, such as Option Grids, which prompt clinicians and patients to consider both risks and benefits of treatment options, are likely to lead to better informed patients and less decision regret. Whilst the Option Grid displays all the treatment options side by side, the verbalization and navigation of the grid was directed by the clinician and the options were intentionally limited on occasion. Previous qualitative work with paediatric rheumatology clinicians found that discussion of treatment options were not only based on clinician preferences but also limited by the clinician's assessment of the clinical situation,^32^ and this also appears to be the case in our study.

Evidence suggests that socially disadvantaged patients are more vulnerable to power imbalances within the clinician patient relationship.^33^ Even patients who are well educated and well informed often struggle to express their knowledge during consultations and participate meaningfully in treatment decisions.^33^ Such power imbalances typically result in patients adopting the role of a passive or compliant patient, a tendency for patients to underplay their knowledge and experiences, and so defer the decision to the clinician whom they see “owns” the knowledge. Although our main trial data indicated that overall in our population of older adults with lower than average health literacy the use of the Option Grid increased levels of SDM, increased patient knowledge and readiness to decide,^16^ in our data we observed some evidence that patients with lower health literacy scores were less positive about being involved in their treatment decision and adopted a fairly passive approach to treatment decision regardless of whether or not they had received an Option Grid. However, there is evidence that many patients feel that they are also not *able* to participate in decision making rather than express that they do not wish to participate.^27^ Using a short decision tool such as an Option Grid is one way of transferring the knowledge ownership and making it explicit to the patient that there is a choice to be made. Clinicians should be mindful that encounter tools such as Option Grids may go some way to empowering and including patients who are disadvantaged by language, low educational attainment and age, but some patients who are most disadvantaged may require additional support in the consultation. This may include spending additional time reading the Option Grid aloud to patients, or encouraging interpreters to do so, or possibly through providing summarized information about treatment options through other visual methods such as simplified images.

We found that many patients wanted other individuals to be involved in treatment decisions. These individuals included primary care physicians, but more commonly patients stated they wanted to discuss their options and preferences with family members. By involving others, patients were drawing on a form of “distributed health literacy”^34^ in which they work collaboratively utilizing the skills of others to help understand health information. This notion also provides support that the Option Grids can be seen not just as a standalone tool for facilitating a conversation within the consultation, but also a tool to be used with the wider family and patient network including primary care physicians.

### 4.4 Conclusions

The findings of this qualitative study, amongst an older patient population who have lower than average health literacy, support the development of Option Grids as a tool to facilitate SDM for patients with osteoarthritis of the knee. The implementation of Option Grids should be considered within routine consultations as patients are supportive of their use.

## References

[1] Elwyn G , Lloyd A , May C , van der Weijden T , Stiggelbout AM , Edwards A . Collaborative deliberation: a model for patient care. Patient Educ Couns. 2014;97:158‐184. 25175366 10.1016/j.pec.2014.07.027

[2] Department of Health . Equity and excellence: liberating the NHS. London; 2010.

[3] Department of Health . Healthy Lives, Healthy People: Our Strategy for Public Health in England. London: The Stationary Office; 2010.

[4] Dagnone T . For Patients' Sake: Patient First Review Commissioner's Report to the Saskatchewan Minister of Health. Saskatchewan: Saskatchewan Ministry for Health; October 2009.

[5] Senate House of Representatives . Patient Protection and Affordable Care Act. 111th Congress, House of Representatives 3590. Washington; 2010.

[6] Stacey D , Légaré F , Col N , et al. Decision aids for people facing health treatment or screening decisions. Cochrane Database Syst Rev. 2014;1. 10.1002/14651858.CD001431.pub4 24470076

[7] Shay L , Lafata J . Where is the evidence? A systematic review of shared decision making and patient outcomes. Med Decis Making. 2015;35:114‐131. 25351843 10.1177/0272989X14551638 PMC4270851

[8] Agoritsas T , Heen A , Brandt L , et al. Decision aids that really promote shared decision making: the pace quickens. BMJ. 2015;350:g7624. 25670178 10.1136/bmj.g7624 PMC4707568

[9] Elwyn G , Lloyd A , Joseph‐Williams N , et al. Option Grids: shared decision making made easier. Patient Educ Couns. 2013;90:207‐212. 22854227 10.1016/j.pec.2012.06.036

[10] Alkan B , Fidan F , Tosun A , Ardiçoğlu Ö . Quality of life and self‐reported disability in patients with knee osteoarthritis. Mod Rheumatol. 2014;1:166‐171. 10.3109/14397595.2013.854046 24261774

[11] Arthritis Research UK . Osteoarthritis in General Practice: Data and Perspectives. London: Arthritis Research UK; 2013.

[12] Culliford D , Maskell J , Beard D , Murray D , Prince A , Arden N . Temporal trends in hip and knee replacement in the United Kingdom 1991 to 2006. J Bone Joint Surg. 2010;92:130‐135. 20044691 10.1302/0301-620X.92B1.22654

[13] Hawker GA , Wright J , Badley E , Coyte P . Perceptions of, and willingness to consider, total joint arthroplasty in a population‐based cohort of individuals with disabling hip and knee arthritis. Arthritis Rheum. 2004;51:635‐641. 15334438 10.1002/art.20524

[14] Caldon L , Collins K , Reed M , et al. Clinicians’ concerns about decision support interventions for patients facing breast cancer surgery options: understanding the challenge of implementing shared decision‐making. Health Expect. 2011;14:133‐146. 21029281 10.1111/j.1369-7625.2010.00633.x PMC5060572

[15] Durand M , Carpenter L , Dolan H , et al. Do interventions designed to support shared decision making reduce health inequalities? A systematic review and meta‐analysis. PLoS One. 2014;9:e94670. 24736389 10.1371/journal.pone.0094670 PMC3988077

[16] Elwyn G , Pickles T , Edwards A , et al. Supporting shared decision making using an Option Grid for osteoarthritis of the knee in an interface musculoskeletal clinic: a stepped wedge trial. Patient Educ Couns. 2016;99:571‐577. 26566194 10.1016/j.pec.2015.10.011

[17] Marrin K , Brain K , Durand M‐A , et al. Fast and frugal tools for shared decision‐making: how to develop Option Grids. Eur J Pers Cent Healthc. 2013;1:240‐245.

[18] National Institute of Social Care and Excellence . Osteoarthritis: Care and Management in Adults: Clinical Guidelines. London: NICE; 2014.

[19] Marrin K , Wood F , Firth J , et al. Option Grids to facilitate shared decision making for patients with Osteoarthritis of the knee: protocol of a single site, efficacy trial. BMC Health Serv Res. 2014;160. 10.1186/1472-6963-14-160 PMC3986464 24708747

[20] Elwyn G , Rasmussen J , Kinsey K , et al. On a learning curve for shared decision making: interviews with clinicians using the knee osteoarthritis Option Grid. J Eval Clin Pract. 2016;. https://doi.org/10.1111/jep.12665. 10.1111/jep.12665 27860101

[21] Wood F , Phillips K , Edwards A , Elwyn G . Working with interpreters: the challenges of introducing Option Grid patient decision aids. Patient Educ Couns. 2017;100:456‐464. https://doi.org/10.1016/j.pec.2016.09.016. pii: S0738‐3991(16)30444‐X [Epub 2016 Sep 22]. 27745941 10.1016/j.pec.2016.09.016

[22] Bass P , Wilson J , Griffith C . A shortened instrument of literacy screening. J Gen Intern Med. 2003;18:1036‐1038. 14687263 10.1111/j.1525-1497.2003.10651.x PMC1494969

[23] Braun V , Clarke V . Using thematic analysis in psychology. Qualitative research in psychology. Qual Res Psychol. 2006;3:77‐101.

[24] Ryan G , Bernard H . Data management and analysis methods In: Denzin N, Lincoln Y, eds. Handbook of Qualitative Research. Thousand Oaks, CA: Sage; 2000.

[25] Office of National Statistics . KS501 Qualifications and Students. Newport, Wales: Office of National Statistics; 2011.

[26] Ahmad N , Ellins J , Krelle H , Lawrie M . Person‐Centred Care: From Ideas to Action: Bringing Together the Evidence on Shared Decision Making and Self‐Management Support. London: The Health Foundation; 2014.

[27] Joseph‐Williams N , Elwyn G , Edwards A . Knowledge is not power for patients: a systematic review and thematic synthesis of patient‐reported barriers and facilitators to shared decision making. Patient Educ Couns. 2014;94:291‐309. 24305642 10.1016/j.pec.2013.10.031

[28] Newsome A , Sieber W , Smith M , Lillie D . If you build it, will they come? A qualitative evaluation of the use of video‐based decision aids in primary care. Fam Med. 2012;44:26‐31. 22241338

[29] Bhavnani V , Fisher B . Patient factors in the implementation of decision aids in general practice: a qualitative study. Health Expect. 2010;13:45‐54. 19811545 10.1111/j.1369-7625.2009.00556.x PMC5060516

[30] Tiedje K , Shippee N , Johnson A , et al. They leave at least believing they had a part in the discussion’: Understanding decision aid use and patient–clinician decision‐making through qualitative research. Med Decis Making. 2013;93:86‐94. 10.1016/j.pec.2013.03.013 PMC3759553 23598292

[31] Fowler F , Gerstein B , Barry M . How patient centred are medical decisions? Results of a National Survey. JAMA Intern Med. 2013;173:1215‐1221. 23712194 10.1001/jamainternmed.2013.6172

[32] Lipstein E , Brinkman W , Sage J , Lannon C , Morgan DeWitt E . Understanding treatment decision making in juvenile idiopathic arthritis: a qualitative assessment. Pediatr Rheumatol. 2013;11:1‐8. 10.1186/1546-0096-11-34 PMC3849714 24079577

[33] Frosch D , May S , Rendle K , Tietbohl C , Elwyn G . Authoritarian physicians and patients’ fear of being labeled “difficult” among key obstacles to shared decision making. Health Aff (Millwood). 2012;31:1030‐1038. 22566443 10.1377/hlthaff.2011.0576

[34] Edwards M , Wood F , Davies M , Edwards A . Distributed health literacy’: longitudinal qualitative analysis of the roles of health literacy mediators and social networks of people living with a long‐term health condition. Health Expect. 2013;16:1180‐1193. 10.1111/hex.12093 PMC5060848 23773311

